# The Dynamic Landscape of Capsid Proteins and Viral RNA Interactions in Flavivirus Genome Packaging and Virus Assembly

**DOI:** 10.3390/pathogens13020120

**Published:** 2024-01-28

**Authors:** Anastazia Jablunovsky, Joyce Jose

**Affiliations:** 1 Department of Biochemistry and Molecular Biology, The Pennsylvania State University, University Park, PA 16802, USA; akj5127@psu.edu; 2 The Huck Institutes of the Life Sciences, The Pennsylvania State University, University Park, PA 16802, USA

**Keywords:** flavivirus, capsid protein, viral RNA, assembly, packaging

## Abstract

The *Flavivirus* genus of the *Flaviviridae* family of enveloped single-stranded RNA viruses encompasses more than 70 members, many of which cause significant disease in humans and livestock. Packaging and assembly of the flavivirus RNA genome is essential for the formation of virions, which requires intricate coordination of genomic RNA, viral structural, and nonstructural proteins in association with virus-induced, modified endoplasmic reticulum (ER) membrane structures. The capsid (C) protein, a small but versatile RNA-binding protein, and the positive single-stranded RNA genome are at the heart of the elusive flavivirus assembly process. The nucleocapsid core, consisting of the genomic RNA encapsidated by C proteins, buds through the ER membrane, which contains viral glycoproteins prM and E organized as trimeric spikes into the lumen, forming an immature virus. During the maturation process, which involves the low pH-mediated structural rearrangement of prM and E and furin cleavage of prM in the secretory pathway, the spiky immature virus with a partially ordered nucleocapsid core becomes a smooth, mature virus with no discernible nucleocapsid. This review focuses on the mechanisms of genome packaging and assembly by examining the structural and functional aspects of C protein and viral RNA. We review the current lexicon of critical C protein features and evaluate interactions between C and genomic RNA in the context of assembly and throughout the life cycle.

## 1. Introduction

Flaviviruses are enveloped, icosahedral viruses, many of which infect humans, causing a broad range of symptoms, including encephalitis, hemorrhagic fever, and microcephaly in infants [1]. Flaviviruses are primarily transmitted to humans by mosquitoes and ticks, although infections with no known vector and human-to-human transmission have been reported [2]. Well-studied mosquito-borne flaviviruses include dengue virus (DENV), Zika virus (ZIKV), yellow fever virus (YFV), West Nile virus (WNV), and Japanese encephalitis virus (JEV), and tick-borne viruses include Powassan virus (POWV) and tick-borne encephalitis virus (TBEV). The *Flavivirus* genus comprises over 70 viruses, all of which have the potential to spill over into human populations [3]. Given the risk of emerging and re-emerging flaviviruses, the urgency to develop effective vaccines and antiviral therapies that target specific viral functions and stop infection at the source is rising. Currently, effective vaccines are available to protect against YFV, TBEV, and JEV [4]. Whereas vaccines targeting DENV, WNV, and ZIKV still face complications due to limited efficacy and antibody-dependent enhancement among related serotypes, as seen in DENV [5,6,7,8]. Several aspects of the flavivirus life cycle remain incompletely understood, posing challenges for antiviral drug development. An ideal antiviral therapy would require simultaneous targeting of multiple viral processes to mitigate the risk of resistance mutations. Therefore, a comprehensive molecular understanding of the lifecycle is essential for the effective combating of flaviviruses.

The flavivirus virion is composed of membrane (M) and envelope (E) transmembrane proteins icosahedrally arranged on an ER-derived lipid bilayer, surrounding a nucleocapsid core (NC) comprising the capsid (C) protein and genomic RNA [9]. The positive single-stranded RNA genome is an 11 kb molecule with 5′ and 3′ untranslated regions flanking a single open reading frame, which encodes three structural proteins [C, precursor membrane protein (prM), and E] and seven nonstructural proteins (NS1, NS2A, NS2B, NS3, NS4A, NS4B, and NS5). During the flavivirus life cycle of an infected cell, initial assembly leads to the formation of an immature virus particle in the ER lumen, which undergoes dramatic low pH-mediated conformational changes and furin processing in the secretory pathway to release the mature virion. Cryo-EM studies in recent years have significantly advanced our knowledge of both immature [10,11,12,13] and mature [13,14,15,16,17] flavivirus structures. This review covers the current understanding of genome packaging and viral particle assembly in flaviviruses by focusing on the interconnected roles of the two major components, C protein and viral RNA.

## 2. Overview of the Flavivirus Life Cycle

**Figure 1:**
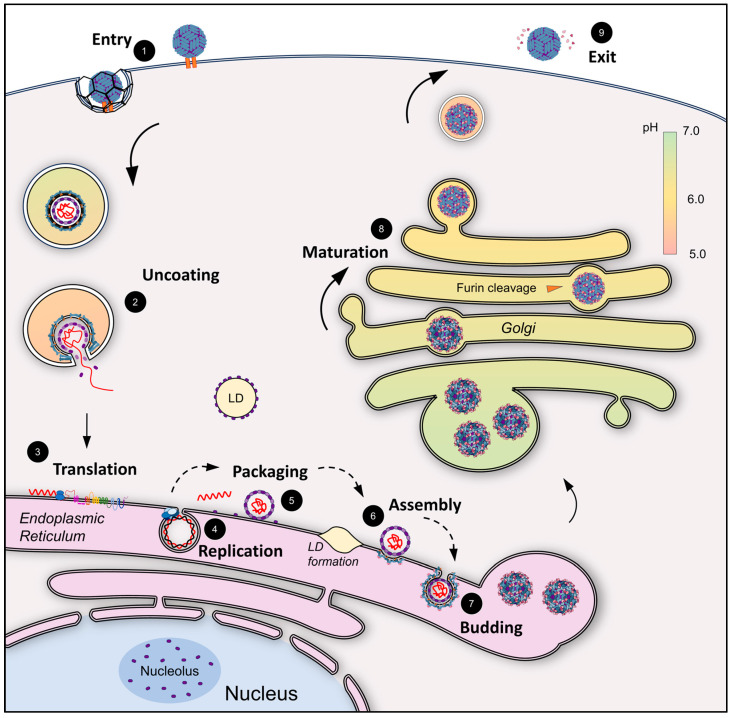
Overview of the flavivirus life cycle. (1) Receptor binding and virus entry via clathrin-mediated endocytosis, (2) fusion of the viral and endosomal membranes followed by uncoating of the viral genomic RNA, (3) translation of viral polyprotein on the ER membrane, (4) replication via the viral replication complex in virus-induced compartments, (5) packaging and NC formation, (6) envelopment of NC and formation of immature particles (dashed arrows between 4 and 6 show proposed steps with unconfirmed mechanisms), (7) immature particles bud into the ER, (8) maturation triggered by low-pH-mediated rearrangements and furin cleavage in the late Golgi, and (9) mature viral particle exit and release of the cleaved pr peptide. C is shown to localize to lipid droplets and the nucleolus.

**Figure 2:**
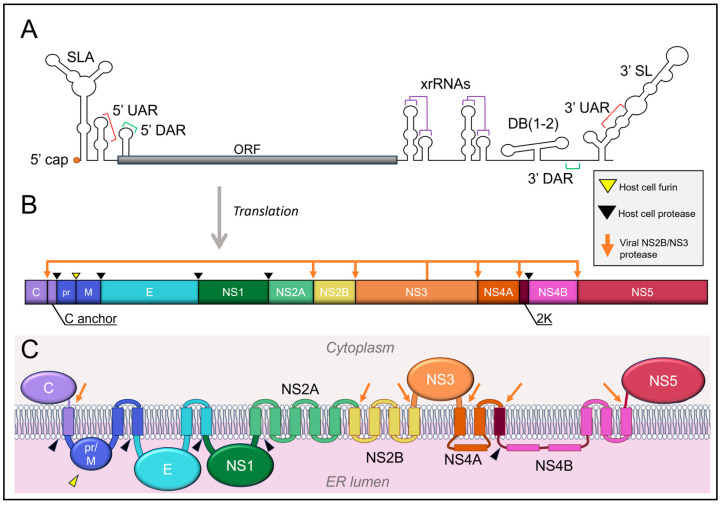
Flavivirus genome organization. Diagrams are not to scale. (**A**) Flavivirus genomic RNA with important secondary UTR structures is shown. (**B**) Polyprotein organization with cleavage sites indicated by arrows. (**C**) Polyprotein topology relative to ER membrane.

Flavivirus infection begins with virus entry when the E glycoprotein interacts with an entry receptor, triggering clathrin-mediated endocytosis, although some flaviviruses, including JEV, may use a clathrin-independent entry mechanism (Figure 1) [18,19,20]. As the compartment progresses from early endosome (pH~7.0) to late endosome (~6.8–5.3), the low pH triggers a conformational change and E dimer dissociation into fusogenic trimers [21,22]. The E protein is a class II membrane fusion protein that exposes an internal fusion loop at low pH that inserts into the endosomal membrane [23,24,25]. This triggers the initiation of membrane fusion, which allows the viral envelope to fuse with the endosomal membrane, and the E protein forms the post-fusion trimers [26,27,28] (Figure 1). The conformational changes of the individual E subunits in the trimer cause inter-bilayer lipid contacts, completing the viral and host membrane merger and fusion pore formation [29], resulting in disassembly and the release of the viral RNA genome into the cytoplasm for translation and replication (Figure 1) [30,31,32]. Translation of viral RNA leads to the co-translational translocation of the highly membrane-associated viral polyprotein into the ER membrane [33,34] (Figure 2). The proteolytic processing of the polyprotein occurs co- and post-translationally by host and viral proteases, leading to the production of ten viral proteins (C, prM, E, and NS1-NS5) (Figure 2). Polyprotein cleavage sites are processed by either host cell proteases or the viral protease NS3 along with NS2B as a cofactor, as indicated in Figure 2 [35,36]. NS2B/NS3 cleaves C from its membrane anchor between the translocation signal helix of C and prM [37]. 

During replication, the viral RNA-dependent RNA polymerase (RdRp) NS5 generates a negative-sense RNA strand from the positive-sense genomic RNA template, and together, these form a double-stranded RNA intermediate, which must be protected from innate host cell dsRNA recognition machinery [38,39,40]. In flaviviruses, protection of the dsRNA replication intermediate is achieved by anchoring the replication machinery in virus-induced invaginations of the ER known as replication compartments [38,41,42] (Figure 1). Flaviviruses induce ER membrane modifications, including vesicle packets (VPs) that are presumed to represent the site of RNA replication and smooth convoluted membranes (CM) that are potential sites of RNA translation and polyprotein processing [41,42]. New positive-strand RNA molecules are synthesized by the replication complex comprising viral proteins, viral RNA, and host factors; the nascent genomic RNA molecules exit the replication complex, most likely through a pore at the neck of the replication compartment [41,42] (Figure 1). The genomic RNA must be transported to serve one of three functions: (i) translation to generate new viral proteins, (ii) formation of a new replication complex, or (iii) assembly of progeny virions. The factors that determine the fate of each positive-strand RNA molecule are not well understood [43]. 

Virus assembly is initiated with the packaging of the positive-strand RNA by C protein [35] (Figure 1). It has been proposed that approximately 120 C proteins (60 C-C dimers) accumulate to form the nucleocapsid core (Figure 1) [9,44]. However, unlike the structurally confirmed T = 3 quasi-icosahedrally arranged 180 copies of transmembrane M and E proteins that form the surface of the virion, the number and arrangement of C proteins within the virion have not been experimentally determined [45]. Currently, the RNA and C protein binding mechanisms are not fully understood from the perspective of either C or RNA. Although RNA binding assays have shown that the 5′UTR of flavivirus RNA can outcompete non-specific RNA interactions with C in the Kunjin (KUNV) strain of WNV, the measured binding affinity is not high enough to be considered a bona fide packaging signal [46]. More recently, the replication protein NS2A has been identified as an RNA chaperone for DENV and ZIKV, potentially assisting nucleocapsid core assembly by interacting with the 3′ UTR of genomic RNA [47,48]. The nucleocapsid core is either concurrently or consecutively enveloped by the transmembrane structural proteins prM and E, along with an ER-derived lipid bilayer, to form the immature virus particle that buds into the ER lumen (Figure 1). The newly generated immature particle is trafficked through the secretory pathway, where a drop in pH leads to a conformational change in the 60 prM/E heterotrimers, which fold down from the immature spiky state to a smooth immature state composed of 90 prM/E heterodimers [10,11,12] (Figure 1). This rearrangement exposes a cleavage site, which is recognized and cleaved by host-cell furin to pr and M proteins, forming the smooth mature virus [49]. After furin cleavage, the pr peptide remains associated with the surface of the smooth mature virus at a neutral pH, preventing premature membrane fusion in the late Golgi [50] (Figure 1). Finally, the mature virus is released into the extracellular milieu via the secretory pathway, at which point the pr peptide dissociates at physiological pH, making the fusion-competent, mature virions poised for subsequent infection. 

## 3. Structural Features of the Capsid Protein

**Figure 3:**
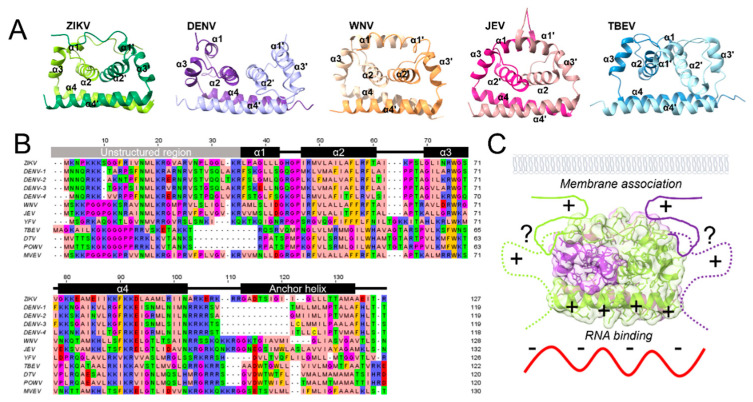
Structures of capsid protein. (**A**) Comparison of flavivirus capsid protein structures from left to right: ZIKV (PDB 5YGH), DENV (PDB 1R6R), WNV (PDB 1SFK), JEV (PDB 5OW2), and TBEV (PDB 5O6V). (**B**) Multiple sequence alignment of flavivirus capsid protein sequences. Secondary structures are denoted by labeled boxes above. (**C**) Proposed interacting regions of capsid (ZIKV structure used as a model). Concentration of positive charge on capsid protein is indicated by + symbol, and negative charge on RNA is indicated by - symbol. The N-terminal region is shown as solid or dashed lines with question marks demonstrating multiple potential conformations.

The flavivirus capsid protein is small (~10–12 kDa), comprising ~100 amino acids known to form a dimer in solution [51,52]. The C protein structures of four mosquito-borne flaviviruses have been solved via X-ray Crystallography (WNV: PDB 1SFK, ZIKV: PDB 5YGH, JEV: PDB 5OW2) and NMR spectroscopy (DENV: PDB 1R6R and ZIKV: PDB 6C44), as well as one tick-borne flavivirus solved by NMR (TBEV: PDB 7YWQ) [52,53,54,55,56,57] (Figure 3A). Although the 3-dimensional structure of the C protein is primarily conserved across flaviviruses [58], it displays only ~55% amino acid sequence conservation, the lowest of all three structural proteins [59]. Multiple sequence alignments of flavivirus C proteins show a high concentration of positively charged residues (Figure 3B). The ~30–100 aa 3D structure of the protein is composed of four α helices (α1–α4), which form three layers. The top layer contains α1 and an N-terminal unstructured domain, which is proposed to be involved in membrane association and RNA binding [60,61]. The middle layer is formed by α2, which is mainly hydrophobic and responsible for dimerization, and α3 and the connecting loop between α2 and α3 [56]. The bottom layer consists of the longest helix, α4, which is proposed to be involved in RNA binding and nuclear localization [46,61]. The length of the unstructured N-terminal region varies widely from TBEV (~18 aa) to ZIKV (~36 aa). Despite their disordered nature, several residues in this domain have been shown to have distinct functions [62]. Positively charged regions are likely involved in RNA binding in tandem with the α4, including two highly conserved residues (K31 and R32 DENV numbering), which, when mutated, were found to impact RNA binding and infectivity in both TBEV and DENV [63,64]. 

The first helix of the C protein, α1, is highly variable in both length (~5–16 aa) and orientation based on the available 3D structures (Figure 3A). The α1 and α1′ helices of the DENV and TBEV C dimers are widely spaced (DENV ~15 Å, TBEV ~20 Å distance), exposing a large hydrophobic cleft. This cleft has been proposed to interact with membranes because it would expose a sizeable hydrophobic area [65]. However, in the dimeric structures of ZIKV, WNV, and particularly JEV, the α1 helices are in close proximity (~9.4, ~8.2, and ~5.7 Å, respectively), effectively closing the cleft and making it inaccessible for membrane interactions. Molecular dynamics simulations have predicted that the flexibility of DENV C in solution might allow switching between the open and closed states of the hydrophobic cleft [66]. Although this flexibility might exist for all C proteins, the hydrophobic cleft is closed in the crystal structures of ZIKV, JEV, and WNV C proteins. It is currently not known how membrane binding would impact this cleft.

The α2 helix (~11–16 amino acids) is predominantly hydrophobic and runs antiparallel along the center of the protein (Figure 3A). Dimerization is driven by hydrophobic interactions between α2 and α4 [51,64,67]. Mutational analysis of α2 hydrophobic clusters has revealed the functional role of F46, L50, F53, and F54 in assembly and residues G42, P43, and R45 in nuclear localization of JEV C protein [63]. The α2 helix also plays a significant role in C localization to cellular membranes. In DENV, mutation of hydrophobic residues F53 and F54 alone was insufficient to alter the lipid droplet (LD) association of C protein, whereas including L46A/L50A mutations along with F53A/L54A prevented C from localizing to LD altogether [64,65]. Residues L50 and L54 were also implicated in the nuclear localization of the C protein [65]. 

The α3 helix (~9–11 amino acids) is oriented perpendicular to the rest of the protein and contains several surface-exposed residues. In the case of the WNV C protein, an infectious virus generated from a 2-plasmid cDNA system to study the impact of the C mutation on virus growth tolerated large deletions of up to 37 residues, some of which eliminated the α3 helix, indicating that it is not a critical region [68,69]. In contrast, a study for developing a live attenuated vaccine candidate for ZIKV [70] found that a deletion mutant virus CΔ63-71 (entire alpha 3 helix) was limited to 1–2 rounds of infection and suggested a possible defect in uncoating. Additionally, in a single-round infectious particle system of JEV, mutation K63A/L66A was found to attenuate virus production [63]. Recent work from our lab used mutational analysis of surface-exposed residues to explore C protein α3 in ZIKV [71]. We detected a significant attenuation in virus production when residue N67 was mutated to alanine, which we attributed to a virus assembly defect. Furthermore, N67A produced a second site reversion in the M protein, implicating a genetic interaction between C and M proteins related to assembly [71].

The fourth helix is on the opposite side of the protein relative to the proposed lipid binding region (α1–α2), and hence it has been suggested to face the interior of the NC, where genomic RNA is presumably located [52,54,72]. Recent studies from DENV and JEV have supported this model and further shown that C can interact simultaneously with membranes and nucleic acids, indicating that the interaction regions on C are structurally distinct [61,73]. While the surface-exposed, positively charged residues on α4 are implicated in RNA binding [46,74], interior-facing hydrophobic residues are important for dimerization [51,64,67]. A recent NMR structure of ZIKV C proteins has revealed multiple dimer-stabilizing salt bridges between E76/R45, D87/R55′, and K75/E79, which were not observed in previous crystal structures [57]. A conserved intermolecular salt bridge has also been identified in DENV, formed by D87/D87′, with a biochemically characterized role in dimerization [75]. A recent study used DENV C protein-mutated residues R85 and R85′ to bind cysteines, forcing a disulfide bond between the two α 4 helices to establish dimerization [76]. This mutation formed a dimer stable enough to maintain core-like structures in vitro, although these cores contained no nucleic acid. Notably, the α4 helix also has multiple canonical nuclear localization signals required for C trafficking to the nucleus in DENV [77,78]. 

The C protein has a C-terminal transmembrane helix that anchors C onto the ER membrane prior to protease cleavage by NS2B/NS3. This C-terminal helix that functions as a translocation signal sequence for prM protein [37] has also been termed α5 by Tan et al., 2020 [44]. Processing the α5 anchor helix requires two distinct, sequential cleavage events, first by host signalase in the ER lumen, followed by NS2B/NS3 viral protease on the cytoplasmic side [79,80]. The temporal control of these events is critical for the efficient formation of infectious viruses, i.e., increasing the efficiency of the second event reduces specific infectivity [81,82]. Based on sequence alignments, the length of the anchor helix varies among flaviviruses from ~15–20 aa. A live-cell imaging study by Gabriel et al., 2020 [83] developed a fluorescently tagged cDNA clone of ZIKV by duplicating C to allow attachment of a tag to one C protein without interfering with the packaging of the other C protein. In this construct, the virus contains one anchorless C and one tagged-anchored C [78,83]. This strategy produced a live infectious virus; however, the virion itself was not fluorescent, indicating that only the untagged C was assembled and the anchor is not required for virus assembly. Additionally, in a pseudo-virus system using ZIKV as a model where prM and E were expressed in trans with either anchored or unanchored C, both C constructs were able to package and assemble into virus particles capable of single-round infection [79]. However, viral particles were not formed when prM was preceded by a nonhomologous anchor, indicating that the anchor processing step is essential for the temporal processing of pr, not C. Although this hydrophobic transmembrane anchor is not included in any available 3D structures of C protein, the C anchor helix has been proposed to be involved in assembly in ZIKV, based on a 9 Å cryo-EM structure of the immature virion in which a low-resolution density was observed between C densities that was interpreted to be the fifth helix [44]. From this data, a subset of C proteins is proposed to be released from the membrane with the uncleaved anchor still intact and hypothetically involved in lateral interactions that form the nucleocapsid core in the case of ZIKV. Models based on this theory suggest that anchored C makes up the exterior of the core, while unanchored C binds RNA in the center [84]. However, as there is no antibody that can distinguish between anchored and unanchored C, the presence of α5 in the purified virus has not been proven biochemically. Furthermore, how the virus would overcome an energetic barrier to remove this anchor from the membrane has not been addressed.

## 4. Functions of Capsid Protein

### 4.1. Packaging

Flavivirus genomic RNA has not been reported to contain a bona fide packaging signal for C protein to specifically bind and package the genomic RNA [9]. However, the C protein is known to bind miscellaneous nucleic acids non-specifically through electrostatic effects, most likely with the backbone phosphates [46]. Despite this feature, C can still exclude single-stranded RNA, double-stranded (ds)RNA, and host RNAs to selectively package only newly synthesized positive-strand genomic RNA [85]. One current theory is that RNA packaging specificity is afforded by NS2A, which is proposed to function as an RNA chaperone. Protein-RNA pulldown analyses have indicated that NS2A binds structures in the 3′UTR in both via residues on an exposed cytosolic loop [48,86]. In the NS2A-mediated assembly model, newly synthesized positive-strand RNA emerging from the replication complex is effectively “passed” by NS2A to the awaiting unprocessed C proteins, which triggers the accumulation of nearby C proteins to form the NC [47].

### 4.2. Core Assembly

**Figure 4:**
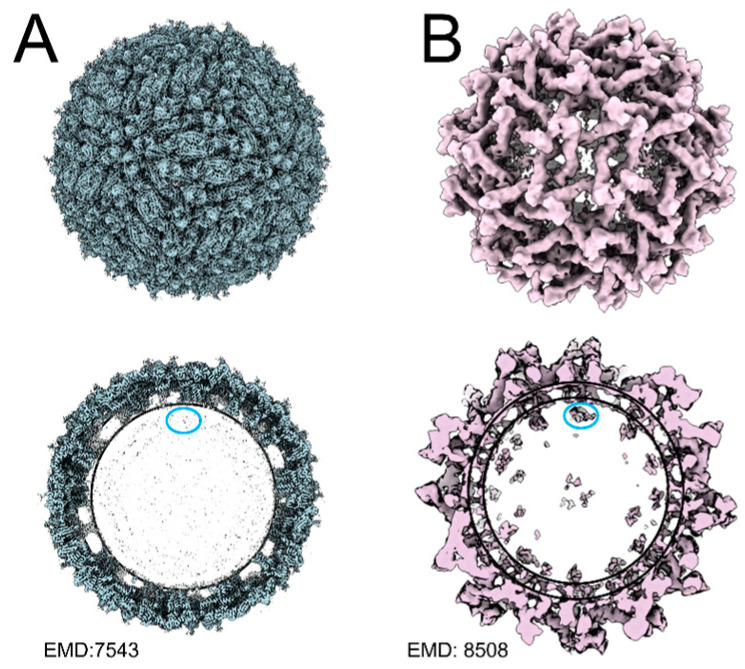
Virion structures of a flavivirus as determined by cryo-EM. (**A**) Structure of mature ZIKV (**top**) and cross-section of mature ZIKV (**bottom**) with the believed location of C protein circled in blue, showing no observable density. (**B**) Structure of immature ZIKV (**top**) and cross-section of immature ZIKV (**bottom**) with believed C protein density circled in blue.

Unlike other enveloped positive-strand RNA viruses, such as alphaviruses, which readily form cores when RNA or single-stranded DNA is introduced to purified capsid proteins [87,88], flavivirus cores are difficult to generate in vitro [64,89]. A study by Kiermayer et al. in 2004 [90] produced core-like particles using TBEV C; however, the size was 2–3 nm larger than the predicted core diameter of ~30 nm. Stabilization of the dimeric structure through the addition of a forced disulfide bond allowed the formation of core-like particles using DENV C. However, these particles contained no RNA [76]. Despite these efforts, no specific residues have been implicated in core formation, and the mechanism of core assembly remains unknown. In cryo-EM structures of mature flaviviruses, including ZIKV, DENV, Spondweni virus, Usutu virus, and WNV, the NC is not observed despite achieving high resolution in the outer envelope proteins (Figure 4A, ZIKV) [13,14,15,91,92]. This indicates that the NC presents inherent asymmetry, or at least differential symmetry, compared to the envelope. In contrast, recent immature structures display an internal density that is not seen in mature structures [10,11,44,45], suggesting that prior to maturation, the NC has a certain level of symmetry that is lost when the virus matures (Figure 4B, ZIKV). Notably, in other immature structures, including the Spondweni virus [13] and the insect-specific Binjari virus [15], the resolution of the viral envelope is high, but the NC remains unresolved. Notably, these reconstructions were generated from immature viral particles selected out of a mature virus preparation, indicating that the NC has already undergone a low pH-induced structural rearrangement independent of the outer prM/E proteins.

### 4.3. Interactions with Host Cell Factors

Most C protein pulldown studies have been performed using expression constructs and have identified host protein interactions with both pro- and anti-viral functions. A summary of these interactions has been reviewed in detail by Sotcheff and Routh 2020 [58]. It is clear that the C protein has functions outside of packaging and assembly, including immune evasion and induction of the ribosomal stress response [63,93,94,95,96]. Additionally, the promiscuous nucleic acid binding of the C protein could result in interactions with host cell RNAs that may explain altered translation patterns in infected cells [97,98]. Flavivirus infection is also known to reduce the formation of stress granules (SGs) by interaction with SG pathway proteins [99,100]. However, it has also been proposed, based on in vitro experiments, that C-RNA binding generates phase-separated structures similar to SGs as an assembly mechanism [73].

Flavivirus C proteins are known to associate with cellular membranes, including nuclear membranes and lipid droplets (LD), both in infected cells and when expressed alone [101,102] (Figure 1). The trafficking mechanism that removes C from the ER to the LD is not known, although cytoplasmic LD localization may be a byproduct of LD biogenesis from ER membranes [103]. It has been proposed that C be sequestered on LD to reduce the local concentration of C in the ER and prevent premature packaging [65]. In the hepatitis C virus (HCV), the core protein is similarly targeted to LD, and mutations that prevent or reduce this interaction have been shown to prevent assembly [104,105]. A similar pathway may also facilitate assembly in flaviviruses. In DENV, residues critical for C-LD binding (L50 and L54) prevented particle production, which indicates a role in assembly supporting this theory [65].

In the nucleus, the C protein has been shown to localize specifically to the nucleolus [71,101,106]. This localization pattern has been reported for ZIKV, WNV, DENV, and JEV C proteins [96,101,107,108]. The α4 helix contains putative nuclear localization signals comprising basic residue clusters that are surface-exposed. Residues K73-K74, R85, and K86 prevented trafficking to the nucleus in DENV when mutated [77]. A live-imaging study using a cDNA clone of DENV containing an mCherry-tagged C protein used cross-correlation analysis to investigate the dynamics of C trafficking and found that C nuclear transport occurs in a bidirectional fashion [78]. The NS5 protein is also observed throughout the nucleus, with implications for host cell translation [108,109]. The C protein localization appears specific to the nuclear membrane and nucleolus, whereas NS5 has been shown to colocalize with small ubiquitin-like modifiers throughout the nucleus [71,110]. Mass spectrometry analyses have demonstrated that the C protein specifically interacts with proteins involved in p53-induced apoptosis, RNA modification, ribosomal stress signaling, and protein turnover in the nucleus [58,94,96,106,111,112]. These functions are less likely to be directly related to virus assembly, which occurs exclusively in association with the ER membrane.

## 5. Important Structural Features of Flavivirus RNA

### 5.1. Circularization and Replication

The flavivirus genome is an ~11 kb positive-sense single-stranded RNA molecule translated upon its release into the cytoplasm following virus entry and disassembly. The 5′ end of the genomic RNA contains a Type 1 (m7GpppNm) cap, which is added by the N-terminal methyltransferase domain of NS5 during replication [113] (Figure 2A). The flavivirus RNA genome does not contain a polyA tail at its 3′ end. The first ~70 nucleotides of the genomic RNA form stem-loop A (SLA), which acts as a promoter recognized by the viral RNA-dependent RNA polymerase NS5 to initiate replication, as recently confirmed by the cryo-EM structure of the RNA-bound NS5 complex [114]. SLA is followed by the much shorter stem-loop B (SLB), which contains the 5′ upstream AUG region (5′UAR) [115,116] (Figure 2A). Flavivirus replication requires pseudo-circularization of the genome, wherein complementary regions in the 5′ and 3′ regions align to form a panhandle-like conformation. The 5′UAR sequence is critical for circularization because it is complementary to the 3′ upstream AUG region in the 3′ UTR (3′UAR) in mosquito-borne flaviviruses, and their role in replication has been demonstrated in DENV, WNV, and KUNV [85,115]. Similarly, there is a downstream AUG region in both the coding region for C (5′DAR) and the 3′ UTR (3′DAR), which align to facilitate circularization. The 5′DAR is located within the RNA sequence that encodes the N-terminal region of C, making it difficult to uncouple replication from C functions [117]. Translation occurs in the linear conformation [118], while replication is limited to the circularized conformation, which has been observed for DENV by atomic force microscopy [115]. The conformational switch from linear to circular is thought to prevent simultaneous processing of the RNA by ribosomes and NS5, which would cause steric collisions inhibiting both processes [119]. It was later confirmed from cryo-electron tomography studies that the replicating RNA is confined to the replication spherules on the ER membrane. In contrast, translation occurs in association with convoluted membranes [41], allowing the spatial separation of replication translation and assembly processes.

### 5.2. Exoribonuclease-Resistant RNAs

A common feature of all known flavivirus infections is the accumulation of RNA fragment molecules 300–500 nt in length and made up of entirely UTR-derived RNA known as subgenomic flavivirus RNA (sfRNA) [120]. The presence of sfRNA in infected cells has been confirmed by a northern blot. The current understanding of sfRNA production and functions has been reviewed extensively [121]. In brief, flavivirus genomic RNA contains one or more exoribonuclease-resistant RNA (xrRNAs) elements in the 3′UTR, which form highly stable pseudoknot structures, including a three-point junction that encircles the RNA strand, protecting it from degradation by host exoribonuclease-1 (XRN1) [122]. Mutations destabilizing the xrRNA structure and allowing degradation to proceed are known to decrease pathogenesis. Although the sequence corresponding to the xrRNA pseudoknots varies among viruses, X-ray crystal structures of xrRNA from ZIKV, Murray Valley encephalitis virus (MVEV), and the lesser-known Tamana bat virus have shown that the secondary structure providing protection from XRN1 is conserved [123,124,125,126].

The potential involvement of sfRNAs in packaging is a matter of debate within the field. Due to the non-specific nature of C-RNA binding, C is not expected to exclude the sfRNA based on size. Furthermore, the 3′UTR element, which is proposed to act as a specificity signal through interaction with NS2A during assembly in ZIKV, is located downstream of the first xrRNA structure, indicating that sfRNAs could be recognized for packaging [47]. Contrary to this notion, the coupling of replication and assembly of flaviviruses may prevent sfRNAs from being packaged. However, the XRN1 enzyme responsible for generating sfRNA is found on the ER and was found to colocalize with replication complexes during WNV infection [127], suggesting that the sfRNA could be produced in the same vicinity as replicating genomic RNA. A study investigating the PR2B strain of DENV-2, which exhibits an increased sfRNA to genomic RNA ratio, detected sfRNA in released particles containing E protein, although it was unclear if these were virus-like particles or virions [128]. The potential benefit of packaging sfRNA along with genomic RNA may be an effective pre-emptive strike against the host cell immune response because sfRNA is involved in pathogenesis, and the initiation of antagonistic interactions prior to replication could give the virus an advantage [128]. Conversely, sfRNA molecules may be “packaged” into structures containing prM/E proteins with no nucleocapsid core, commonly known as subviral particles. These could act as defective interfering particles, antagonizing the immune response of nearby cells and paving the way for the advanced spread of infection.

### 5.3. Post-Transcriptional Modifications

The entire length of the flavivirus positive-strand RNA genome is enriched with post-transcriptional modifications (PTMs) [129,130,131]. A study comparing multiple positive-strand RNA viruses, including DENV, ZIKV, and HCV, examined total viral RNA isolated from infected cells by affinity capture and mass spectrometry to determine overall PTM content [129]. The study found that all viruses sampled contained 30–40 unique PTMs in significant abundance. The RNA modifications identified included well-characterized PTMs such as N6-methyladenosine (m6A) [132] and pseudouridine (ψ) [133], as well as more recently discovered modifications like 5-methoxycarbonylmethyluridine (mCm5U) [134]. A diverse array of reader/writer/eraser enzymes are required to coordinate RNA modifications, and it is not currently known when these interactions occur in flaviviruses. Thus far, the location of RNA modifications has not been mapped to specific sites on the genome or time points in the life cycle. As such, it is not known if genomic RNA receives a uniform modification pattern or if there are differences between individual molecules. RNA modification is known to alter secondary structures and can also impact the binding affinity of RNA-binding proteins [135]. In theory, distinctive modification patterns could impact protein binding and offer a potential mechanism for the differential trafficking of positive-strand viral RNA molecules destined for translation, replication, or virion assembly [43].

## 6. Functions of RNA

### 6.1. Replication Coupled Assembly

The processes of replication and assembly are thought to be tightly coupled in flaviviruses. Transfection of a full-length cDNA clone of KUNV was only able to package RNA when NS5 was functional, despite the continuous generation of viral RNA by the host cell [85]. This indicates that replication by NS5 is a prerequisite to assembly and suggests a mechanism of specificity that excludes non-replicating RNAs. Transmission electron tomography analysis of DENV-infected cells provided the first 3D evidence of replication compartment organization and indicated the presence of a pore at the base of the replication compartment that may facilitate the extrusion of the genomic RNA post-replication [41]. Additionally, virus assembly structures were observed in close proximity to replication compartments, indicating a spatial component to packaging specificity, although the direct transport of viral RNA from replication to the assembly site has not been observed. Similar data were later obtained for other flaviviruses, including ZIKV [42] and TBEV [136], as well as DENV-infected mosquito cells [137]. Additionally, trans-encapsidation experiments in which structural proteins are co-transfected with non-structural proteins have shown that newly generated replicon RNA does not need to be packaged by cis-acting C/prM/E proteins for WNV, DENV, and JEV [138,139]. In fact, due to the nonspecific nature of C-RNA binding, chimeric virus particles can be generated with viral RNA from one virus and prM/E structural proteins from another as long as the RNA is newly replicated [140]. For example, JEV replicons have been shown to form single-round infectious particles when paired with prM/E structural proteins from WNV, YFV, and TBEV, although the efficacy varies [138].

### 6.2. Interactions with Host-Cell Proteins

Flaviviruses are known to hijack host proteins at all stages of the virus life cycle, and viral RNA is no exception [141,142]. It follows that host cell factors may be critical components of virus assembly. The protein interactome of flavivirus RNA has been investigated by coimmunoprecipitation studies and pulldown experiments using aptamers. RNA affinity capture of biotinylated viral RNA fragments followed by mass spectrometry has found that SND1, a protein with known RNA transport function, binds to the 3′UTR of DENV as well as proteins involved in RNA splicing and stability [143]. Comprehensive immunoprecipitation of RNA in ZIKV- and DENV-infected human cells followed by quantitative LC/MS/MS has recovered over 400 RNA-interacting proteins, including both viral and host factors [144]. Viral RNA from DENV fused with an S1 streptavidin-binding aptamer was used for endogenous RNA pulldown, although the aptamer-fused clone was unable to produce an infectious virus; therefore, all proteins were identified from transfected cells [145]. Notably, the majority of the selected proteins of interest for these studies were connected to replication or translation, not virus assembly.

### 6.3. Intracellular Dynamics of Flaviviral RNA

Interactions between RNA and the host cell could also be investigated via microscopy. However, visualization of flavivirus genomic RNA to date has been elusive due to resolution constraints and limitations in RNA tagging [43]. Fluorescence in situ hybridization (FISH) experiments, as well as immunofluorescence assays using antibodies against dsRNA, have revealed the localization of viral RNA in fixed cells [100,146,147,148]. The colocalization of TBEV RNA with mRNA trafficking proteins in infected neuronal cells, which may be linked to neuropathic effects caused by the virus, was determined by FISH [149]. However, thus far, the resolution in RNA FISH imaging has not been sufficient to differentiate individual molecules, which is necessary to determine post-replication RNA trafficking spatially.

A major disadvantage of fixed-cell imaging is the lack of temporal information obtained from a live infected cell, including real-time information on LD structures and cell dynamics [150]. Aptamer tagging is an alternative to hybridization for RNA imaging that involves the insertion of an RNA structure that binds to a fluorescent reporter molecule, such as a dye. This method has proven difficult in flaviviruses because successful replication is highly dependent on secondary RNA structures for circularization [115], and most aptamer-based systems require large inserts that can disrupt these structures, resulting in non-replicating viruses [43,151]. Replicon RNA of TBEV has been successfully tagged with the aptamer MS2, enabling tracking of the positive-strand RNA dynamics at a modest resolution [151]. Based on photobleaching experiments, it was found that RNA populations are not continuously diffused but contain some immobile fraction that is arrested by either protein binding or some other spatial constraint [151]. In other viruses, including Sindbis virus, coxsackievirus, and HCV, high-resolution imaging of viral RNA in live cells has led to a breakthrough understanding of RNA trafficking and infection dynamics [152,153,154,155]. Single-molecule imaging of mRNAs has also led to significant advances in understanding cellular RNA trafficking [156]. Adopting similar approaches using flavivirus RNA will help to elucidate the connection between replication and assembly.

## 7. Critical Gaps in C-RNA Interactions

A crucial step after virion entry is the release of genomic RNA into the cytoplasm. The disassembly of the nucleocapsid core, often referred to as uncoating, requires C to unbind from viral RNA and is not a well-understood process. In many viruses, including influenza and the Herpes simplex virus, this stage represents a critical bottleneck in the life cycle because genome release that is too early subjects it to degradation, while releasing it too late could interfere with translation [157,158]. In flaviviruses, this constraint is partly achieved through pH control, wherein mature smooth M/E heterodimers can rearrange into fusion trimers at a threshold pH of 6.8, which prevents premature fusion in the early endosome [26]. It is currently unclear whether the pH changes affect the structural arrangement of the core during entry. Post-uncoating studies using YFV and DENV have suggested that ubiquitination of the C protein is a required step in releasing the genomic RNA [74,141,159,160]. It is unclear if the C-RNA interaction is disrupted before or during degradation. Notably, the lack of NC density observed in cryo-EM reconstructions of mature flaviviruses indicates that the core does not have detectable symmetry in mature infectious viruses (Figure 4A) [11,14]. This lack of symmetry may be due to a loss of stabilizing interactions in the NC, which may facilitate genomic RNA release during disassembly. Careful interrogation of C and genomic RNA interactions at very early stages of virus entry will need to be explored to uncover the disassembly mechanisms.

The lack of a packaging signal in flavivirus RNA has left the mechanism of genome encapsidation obscure. The post-replication RNA trafficking process that determines the fate of a single RNA molecule for translation, replication, or packaging is inadequately understood [43,151]. Recent focus has narrowed on NS2A as a potential RNA chaperone [47,48], which may connect packaging specificity with replication-coupled assembly. However, as a membrane protein, the structure of NS2A has not been solved, and the potential cooperative dynamics of RNA binding required to facilitate assembly with C protein are not known. Incidentally, our understanding of C-RNA binding is incomplete from the C-protein perspective. While mutagenesis studies have implicated both α4 and the N-terminal domain, a bona fide structure of C bound to RNA has yet to be solved. Higher-resolution cryo-EM structures of immature viruses may reveal residues involved in interactions between C and prM/E. Moving forward, the molecular details of flavivirus assembly will most likely be discerned by a combination of structure-function studies, including cryo-EM structure of viruses, viral proteins, RNA protein complexes, and cryo-electron tomography of cells infected with wild-type and mutant viruses that are defective in virus assembly processes. With the current danger of both emerging and reemerging flaviviruses worldwide, the pressure to develop safe and effective antiviral strategies persists. Defining the mechanism of flavivirus assembly on a molecular level will pave the way for broadly effective anti-flaviviral drugs, which could be the key to combating these devastating viruses.
